# The predictive value of the systemic immune-inflammation index in chronic thromboembolic pulmonary hypertension

**DOI:** 10.1097/MD.0000000000049689

**Published:** 2026-07-03

**Authors:** Hatice Solmaz, Burcu Uludag, Ayse Colak

**Affiliations:** a Department of Cardiology, University of Health Sciences, Izmir City Hospital, Izmir, Turkey; b Department of Cardiology, Dokuz Eylul University Faculty of Medicine, Izmir, Turkey.

**Keywords:** chronic thromboembolic pulmonary hypertension, inflammation, pulmonary embolism, systemic immune-inflammation index

## Abstract

The systemic immune-inflammation index (SII) is associated with the severity and progression of vascular disorders. However, the relationship between SII and chronic thromboembolic pulmonary hypertension (CTEPH) remains unclear. This study aimed to investigate for the first time the predictive value of SII for the development of CTEPH in patients with a history of unprovoked acute pulmonary embolism (PE). This retrospective case-control study included 62 patients with unprovoked acute PE for the first time. Thirty patients with CTEPH and 32 without CTEPH were compared in terms of baseline and follow-up clinical, echocardiographic, and laboratory parameters obtained at least 3 months after the index event. Univariate and multivariate logistic regression analyses were performed to determine predictors of CTEPH. The diagnostic performance of the SII was evaluated using receiver operating characteristic curve analysis. Among the baseline laboratory parameters related to SII, only platelet count (308 [237–339] vs 259 [207–283], *P* = .028) was significantly higher in the CTEPH group, whereas SII did not differ significantly between the groups. During follow-up beyond 3 months, neutrophil count (5.4 [3.6–6.4] vs 4 [3.3–4.8], *P* = .01), platelet count (313 [219–376] vs 251 [201–295], *P* = .02), and SII (1614 [607–2147] vs 566 [417–727], *P* < .001) were significantly higher, whereas lymphocyte count (1.4 ± 0.5 vs 1.9 ± 0.5, *P* < .001) was significantly lower in the CTEPH group. Multivariate logistic regression analysis demonstrated that the SII was an independent predictor of CTEPH (odds ratio 1.003 [1.000–1.005], *P* = .02). Receiver operating characteristic curve analysis showed that the SII had moderate diagnostic performance for predicting CTEPH (area under the curve = 0.797; 95% confidence interval, 0.682–0.912; *P* < .001), with an optimal cutoff value of 626.7. The SII may represent a practical and easily accessible biomarker for identifying patients at risk of developing CTEPH during follow-up after the first episode of unprovoked acute PE.

## 1. Inroduction

Chronic thromboembolic pulmonary hypertension (CTEPH) is a precapillary form of pulmonary hypertension (PH) that may be curable through surgical intervention.^[1]^ It is generally regarded as a long-term consequence of pulmonary embolism (PE) or venous thromboembolism arising from 1 or more thromboembolic events, regardless of whether these events cause clinical symptoms at the time.^[2]^ Despite the relatively high incidence of acute embolic obstructions, only a small subset of patients progress to CTEPH, and the reasons for this selective progression remain unclear.^[3]^ Previous studies have indicated that inflammation significantly contributes to the development of CTEPH.^[4–6]^ Meanwhile, a recently introduced biomarker, the systemic immune-inflammation index (SII), which is calculated using platelet, neutrophil, and lymphocyte counts, has been applied to explore the association between inflammatory processes and various diseases.^[7–13]^

Although there is growing interest in inflammatory biomarkers, the potential role of SII in predicting the development of CTEPH is not well established. Therefore, the aim of the present study was to evaluate the predictive value of the SII in patients with a history of first-time unprovoked acute PE. To the best of our knowledge, this is one of the first studies to investigate the relationship between the SII and the development of CTEPH.

## 2. Materials and methods

In this retrospective case-control study, 74 patients (33 with CTEPH and 41 non-CTEPH) aged ≥ 18 years with a history of first-time unprovoked acute PE between 2017 and 2025 were initially screened through hospital records and the national e-Nabiz system. However, 12 patients were excluded due to missing variables required for evaluation. Finally, data from 62 eligible patients were analyzed; 30 of whom had already been diagnosed with CTEPH. The diagnosis of acute PE was confirmed by computed tomography pulmonary angiography in patients whose symptoms appeared within 14 days prior to diagnosis. All patients had received at least 3 months of effective anticoagulant therapy. Among them, 32 patients with normal right heart function and normal pulmonary artery pressure on echocardiography were included in group 1, while 30 patients who had developed CTEPH comprised group 2.

The diagnosis of CTEPH was based on imaging findings and hemodynamic data obtained via cardiac catheterization in accordance with the 2022 PH Guidelines from the European Society of Cardiology/European Respiratory Society.^[14]^ The criteria included, after at least 3 months of effective anticoagulation: resting mean pulmonary artery pressure ≥ 21 mm Hg; normal pulmonary artery wedge pressure ≤ 15 mm Hg; elevated pulmonary vascular resistance ≥ 2 Wood units; and persistent PE confirmed by imaging modalities (including ventilation/perfusion [V/Q] scans demonstrating mismatched perfusion defects and evidence of organized, fibrotic clots on computed tomography pulmonary angiography or digital subtraction angiography). Patients with conditions that could influence the SII levels were excluded. These conditions included left heart failure, systemic inflammatory diseases, collagen vascular diseases, acute or chronic infections, unstable coronary syndromes, recent surgery (< 3 months) or trauma, active or previous malignancy, thrombophilia, hematological diseases requiring immunosuppressive therapy, and renal or hepatic failure. Several measures were taken to minimize potential sources of bias. Both the CTEPH and non-CTEPH groups were derived from the same underlying population of patients with first-time unprovoked acute PE. The non-CTEPH group was selected consecutively using predefined inclusion and exclusion criteria. Group classification was based solely on the objective development of CTEPH during follow-up. All clinical, laboratory, and echocardiographic data were collected retrospectively from medical records. These methodological considerations help reduce the likelihood of selection and measurement biases.

Baseline blood samples were obtained from all patients on admission, before treatment initiation. Blood samples obtained after 3 months were also included in the analysis. Routine hematological and biochemical parameters, including N-terminal pro-brain natriuretic peptide (NT-proBNP), high-sensitivity troponin I (hs-TnI), and D-dimer levels, were evaluated. Complete blood count analyses were performed using a UniCel DxH 800 hematology analyzer (Beckman Coulter). Biochemical analyses were performed using an AU 5800 chemistry analyzer (Beckman Coulter). hs-TnI measurements were performed using an ADVIA Centaur XP immunoassay analyzer (Siemens Healthineers). D-dimer levels were measured using a CS-2500 automated coagulation analyzer (Sysmex Corporation).

The SII was calculated using the following formula: SII = platelet count × neutrophil count/lymphocyte count.^[15]^ In addition, the simplified PE severity index (sPESI) was calculated according to the 2019 European Society of Cardiology PE guidelines.^[16]^

No formal sample size calculation was performed prior to the study, as all eligible patients who met the inclusion criteria during the study period were included. A post hoc power analysis based on the observed difference in SII between the CTEPH and non-CTEPH groups indicated that a minimum of 26 patients per group would be sufficient to detect the observed effect size with 80% power at a significance level of 0.05.

The study protocol was approved by the local ethics committee and was conducted in accordance with the principles of the Declaration of Helsinki. Ethical approval for this study was obtained from the Non-Interventional Clinical Research Ethics Committee of Izmir City Hospital (decision number: 2024/112). The requirement for informed consent was waived because of the retrospective design of the study.

## 3. Data analysis

Data analysis was conducted using Statistical Package for the Social Sciences version 27 (SPSS Inc.). The normality of continuous variables was confirmed using histograms and the Kolmogorov–Smirnov test. Continuous data are presented as the median (interquartile range) and mean ± standard deviation. Three statistical tests were applied to compare groups: the chi-square test, Mann–Whitney *U* test, and Student *t*-test, as appropriate. We calculated 95% confidence intervals (CIs) for odds ratios (ORs) using both univariate and multivariate logistic regressions. Clinically relevant variables that were significant in the univariate analysis were included in multivariate analysis. Prior to the multivariate analysis, we checked for multicollinearity to ensure that highly correlated or redundant independent variables were not included. The ability of SII to predict CTEPH was assessed using receiver operating characteristic (ROC) curves, and the area under the curve (AUC) was calculated. Statistical significance was defined as a *P* value < .05.

## 4. Results

We retrospectively analyzed 62 patients who presented with unprovoked acute PE. The CTEPH group included 30 patients who had already been diagnosed and were under regular follow-up, while the non-CTEPH group comprised 32 patients who did not develop CTEPH and were selected retrospectively from the same population. The median time to the second examination, based on clinical, laboratory, and echocardiographic data (excluding catheterization findings), was 98.5 days. The baseline characteristics of all patients and a comparison between the CTEPH and non-CTEPH groups are presented in Table 1. The mean age of all patients was 64.2 ± 16.8 and 50% (n = 31) of the patients were women. Among baseline characteristics, the mean age (69 ± 14 vs 59.7 ± 18.1, *P* = .027) and the sPESI score (4.9 [1–3] vs 1.25 [0–2], *P* = .023) were significantly higher in CTEPH patients than in non-CTEPH patients.

**Table 1:** Baseline characteristics of all patients and comparison between CTEPH and non-CTEPH groups.

	All patients (n = 62)	CTEPH (n = 30)	Non-CTEPH (n = 32)	*P* value
Age* (yrs)	64.2 ± 16.8	69 ± 14	59.7 ± 18.1	.027
Women, n (%)	31 (50)	16 (53.3)	15 (46.9)	.44
BMI† (kg/m^2^)	27.3 (24.8–28.2)	26.4 (24.1–27.5)	28.2 (25.2–31)	.17
Hypertension, n (%)	26 (41.9)	12 (40)	14 (43.8)	.76
Diabetes mellitus, n (%)	13 (21)	8 (26.7)	5 (15.5)	.28
COPD, n (%)	0 (0)	0 (0)	0 (0)	NA
CAD, n (%)	6 (9.7)	4 (13.3)	2 (6.3)	NA
CVD, n (%)	1 (1.6)	1 (3.3)	0 (0)	NA
Smoking status, n (%)				0.15
Smoker	21 (33.9)	8 (26.7)	13 (40.6)	
Ex-smoker	16 (25.8)	11 (36.7)	5 (15.6)	
Never smoked	25 (40.3)	11 (36.7)	14 (43.8)	
sPESI	3 (1–3)	4.9 (1–3)	1.25 (0–2)	.023

BMI = body mass index, BP = blood pressure, CAD = coronary artery disease, COPD = chronic obstructive pulmonary disease, CTEPH = chronic thromboembolic pulmonary hypertension, CVD = cerebrovascular disease, IQR = interquartile range, n = number of patients, NA = not applicable, SD = standard deviation, sPESI = simplified pulmonary embolism severity index.

* Mean ± SD.

† Median (IQR).

The comparison of baseline clinical, echocardiographic, and laboratory data between the CTEPH and non-CTEPH groups is presented in Table 2. Among the baseline echocardiographic data, right ventricular diameter (29.8 [28.7–31.2] vs 26.1 [23–28], *P* < .001), tricuspid regurgitation jet velocity (3.5 [3.3–3.7] vs 3 [2.6–3.5], *P* < .001), and systolic pulmonary artery pressure (sPAP) (54.6 ± 8.6 vs 44.6 ± 14.1, *P* = .02) were significantly higher in the CTEPH group. However, tricuspid annular plane systolic excursion (TAPSE) (17.3 [15.7–18] vs 19.2 [16.2–22], *P* = .012) and TAPSE/sPAP ratio (0.33 [0.25–0.38] vs 0.49 [0.31–0.65], *P* = .002) were significantly higher in the non-CTEPH group. Among the baseline laboratory data, platelet count (308 [237–339] vs 259 [207–283], *P* = .028) and NT-proBNP levels (1736 [614–1815] vs 1155 [127–693], *P* < .001) were significantly higher in the CTEPH group. Among baseline clinical data, median oxygen saturation was significantly lower (89 [87–92] vs 92 [89–95], *P* < .001) in the CTEPH group.

**Table 2:** The comparison of baseline clinic, echocardiographic and laboratory parameters in CTEPH and non-CTEPH groups.

	CTEPH (n = 30)	Non-CTEPH (n = 32)	*P* value
Echocardiographic data			
RV diameter† (mm)	29.8 (28.7–31.2)	26.1 (23–28)	< .001
TR jet velocity† (m/s)	3.5 (3.3–3.7)	3 (2.6–3.5)	< .001
TAPSE† (mm)	17.3 (15.7–18)	19.2 (16.2–22)	.012
sPAP* (mm Hg)	54.6 ± 8.6	44.6 ± 14.1	.02
TAPSE/sPAP† (mm/mm Hg)	0.33 (0.25–0.38)	0.49 (0.31–0.65)	.002
LVEF† (%)	56.6 (55–60)	57.3 (55–60)	.39
Laboratory data			
Hemoglobin* (g/dL)	12.8 ± 2.4	12.6 ± 2.1	.8
WBC count* (x10^9^/L)	8.9 ± 2.7	9 ± 2.8	.9
Lymphocyte count* (x10^9^/L)	1.6 ± 0.6	1.7 ± 0.6	.26
Neutrophil count* (x10^9^/L)	6.3 ± 2.3	6.4 ± 2.8	.9
Platelet count† (x10^9^/L)	308 (237–339)	259 (207–283)	.028
hs-TnI† (ng/mL)	55.8 (12.7–46.2)	38 (8.8–29.5)	.09
NT-proBNP† (pg/mL)	1736 (614–1815)	1155 (127–693)	< .001
D-dimer† (ng/mL)	6121 (2186–9887)	5964 (2237–6776)	.69
CRP† (mg/L)	6 (0.9–7.3)	4.2 (1.2–5.4)	.9
SII†	1625 (653–1941)	1056 (505–1327)	.08
Clinical data			
SBP† (mm Hg)	113 (93–131)	113 (105–121)	.86
DBP† (mm Hg)	71 (62–77)	71 (65–77)	.61
Heart rate† (bpm)	104 (88–119)	98 (88–111)	.16
O_2_ saturation† (%)	89 (87–92)	92 (89–95)	< .001

CRP = C-reactive protein, CTEPH = chronic thromboembolic pulmonary hypertension, DBP = diastolic blood pressure, hs-TnI = high sensitive troponin I, IQR = interquartile range, LVEF = left ventricular ejection fraction, n = number of patients, NT-proBNP = N-terminal pro-brain natriuretic peptide, O_2_ = oxygen, RV = right ventricle, S’ = systolic velocity, SBP = systolic blood pressure, SD = standard deviation, SII = systemic immun inflammatory index, sPAP = systolic pulmonary artery pressure, TAPSE = tricuspid annular plane systolic excursion, TR = tricuspid regurgitation, WBC = white blood cell.

* Mean ± SD.

† Median (IQR).

After 3 months, clinical, echocardiographic, laboratory, and hemodynamic data in the CTEPH and non-CTEPH groups are presented in Table 3. After 3 months, echocardiographic data revealed that right ventricular diameter (33.2 [28.7–36.5] vs 25.2 [23–28], *P* < .001), tricuspid regurgitation jet velocity (3.78 ± 0.47 vs 2.67 ± 0.36, *P* < .001), and sPAP (54.6 ± 8.6 vs 44.6 ± 14.1, *P* = .02) were significantly higher in the CTEPH group. However, the TAPSE (16.9 [14–19] vs 20.8 [19–23], *P* < .001), TAPSE/sPAP ratio (0.28 ± 0.11 vs 0.65 ± 0.22, *P* = .002), and left ventricular ejection fraction (58.1 [55–60] vs 60.3 [60–60.7], *P* = .004) were significantly lower in the CTEPH group.

**Table 3:** Clinical, echocardiographic, laboratory, and hemodynamic parameters after 3 months in the CTEPH and non-CTEPH groups.

	CTEPH (n = 30)	Non-CTEPH (n = 32)	*P* value
Echocardiographic data			
RV diameter† (mm)	33.2 (28.7–36.5)	25.2 (23–28)	< .001
TR jet velocity* (m/s)	3.78 ± 0.47	2.67 ± 0.36	< .001
TAPSE† (mm)	16.9 (14–19)	20.8 (19–23)	< .001
sPAP† (mm Hg)	66 (49–80)	34.5 (29–39)	< .001
TAPSE/sPAP* (mm/mm Hg)	0.28 ± 0.11	0.65 ± 0.22	.002
LVEF† (%)	58.1 (55–60)	60.3 (60–60.7)	.004
Laboratory data			
Hemoglobin* (g/dL)	12.5 ± 2	13 ± 1.6	.22
WBC count† (x10^9^/L)	7.8 (6.4–8.4)	6.7 (5.4–7.8)	.051
Lymphocyte count* (x10^9^/L)	1.4 ± 0.5	1.9 ± 0.5	< .001
Neutrophil count† (x10^9^/L)	5.4 (3.6–6.4)	4 (3.3–4.8)	.01
Platelet count† (x10^9^/L)	313 (219–376)	251 (201–295)	.02
hs-TnI† (ng/mL)	20 (10.7–26.2)	11 (5.7–13.2)	< .001
NT-proBNP† (pg/mL)	1554 (370–1729)	360 (74–194)	< .001
D-dimer† (ng/mL)	1138 (475–1270)	503 (235–754)	.006
CRP† (mg/L)	5.37 (0.4–6.1)	2.94 (0.6–3.8)	.03
SII†	1614 (607–2147)	566 (417–727)	< .001
Hemodynamic data			
MPAP* (mm Hg)	38.5 ± 11.8	-	-
PCWP* (mm Hg)	8.5 ± 2.2	-	-
PVR† (Wood Unit)	8.6 (5.8–9.7)	-	-
Clinical data			
SBP* (mm Hg)	120.2 ± 15.2	125.7 ± 10.5	.1
DBP* (mm Hg)	74.7 ± 9.8	79.3 ± 6.9	.03
Heart rate† (bpm)	89 (81–96)	84 (78–89)	.004
O_2_ saturation† (%)	90.9 (89–93.2)	95.1 (94–97)	<.001

CRP = C-reactive protein, CTEPH = chronic thromboembolic pulmonary hypertension, DBP = diastolic blood pressure, hs-TnI = high sensitive troponin I, IQR = interquartile range, LVEF = left ventricular ejection fraction, MPAP = mean pulmonary artrey pressure, n = number of patients, NT-proBNP = N-terminal pro-brain natriuretic peptide, O_2_ = oxygen, PCWP = pulmonary capillary wedge pressure, PVR = pulmonary vascular resistance, RV = right ventricle, S’ = systolic velocity, SBP = systolic blood pressure, SD = standard deviation, SII = systemic immun inflammatory index, sPAP = systolic pulmonary artery pressure, TAPSE = tricuspid annular plane systolic excursion, TR = tricuspid regurgitation, WBC = white blood cell.

* Mean ± SD.

† Median (IQR).

After 3-month laboratory data showed that neutrophil count (5.4 [3.6–6.4] vs 4 [3.3–4.8], *P* = .01), platelet count (313 [219–376] vs 251 [201–295], *P* = .02), hs-TnI level (20 [10.7–26.2] vs 11 [5.7–13.2], *P* < .001), NT-proBNP level (1554 [370–1729] vs 360 [74–194], *P* < .001), D-dimer level (1138 [475–1270] vs 503 [235–754], *P* = .006), C-reactive protein level (5.37 [0.4–6.1] vs 2.94 [0.6–3.8], *P* = .03), and SII (1614 [607–2147] vs 566 [417–727], *P* < .001) were higher, and lymphocyte count (1.4 ± 0.5 vs 1.9 ± 0.5, *P* < .001) was lower in the CTEPH group compared to the non-CTEPH group.

After 3-month clinical data showed that diastolic blood pressure (74.7 ± 9.8 vs 79.3 ± 6.9, *P* = .03) and oxygen saturation (90.9 [89–93.2] vs 95.1 [94–97], *P* < .001) were lower, and heart rate (89 [81–96] vs 84 [78–89], *P* = .004) was higher in the CTEPH group.

Among clinical and laboratory variables, age (OR 1.037 [1.003–1.073], *P* = .03), sPESI (OR 1.669 [1.042–2.673], *P* = .03), heart rate (OR 1.098 [1.023–1.179], *P* = .01), oxygen saturation (OR 0.626 [0.479–0.819], *P* < .001), SII (OR 1.003 [1.001–1.005], *P* = .002), hs-TnI (OR 1.084 [1.020–1.152], *P* = .009), NT-proBNP (OR 1.001 [1.000–1.002], *P* = .02), and D-dimer (OR 1.001 [1.000–1.003], *P* = .02) were significant determinants of CTEPH (Table 4). Additionally, SII was found to be an independent significant determinant of CTEPH in multivariate logistic regression analysis (OR 1.003 [1.000–1.005], *P* = .02).

**Table 4:** Univariate and multivariate determinants of CTEPH.

Variable	Univariate logistic regression analysis			Multivariate logistic regression analysis		
	OR	95% CI	*P* value	OR	95% CI	*P* value
Age	1.037	1.003–1.073	.03	-	-	-
sPESI	1.669	1.042–2.673	.03	-	-	-
Heart rate	1.098	1.023–1.179	.01	-	-	-
O_2_ saturation	0.626	0.479–0.819	< .001	-	-	-
SII	1.003	1.001–1.005	.002	1.003	1.000–1.005	.02
hs-TnI	1.084	1.020–1.152	.009	-	-	-
NT-proBNP	1.001	1.000–1.002	.02	-	-	-
D-dimer	1.001	1.000–1.003	.02	-	-	-

CI = confidence interval, CTEPH = chronic thromboembolic pulmonary hypertension, hs-TnI = high sensitive troponin I, NT-proBNP = N-terminal pro-brain natriuretic peptide, OR = odds ratio, SII = systemic immun inflammatory index, sPESI = simplified pulmonary embolism severity index.

**Figure 1.**
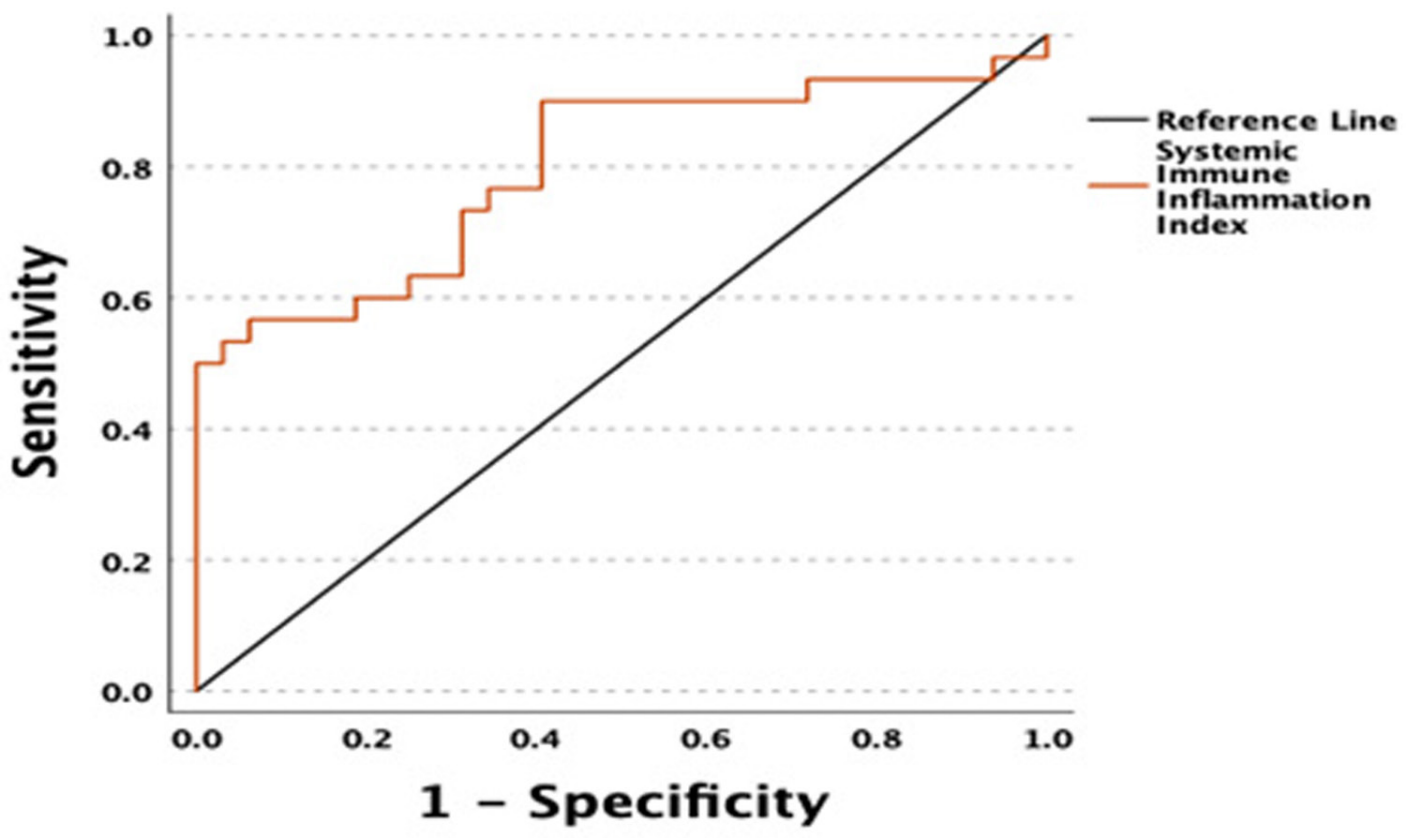
Receiver-operator-curve analysis of the SII for the prediction of CTEPH. CTEPH = chronic thromboembolic pulmonary hypertension, SII = systemic immune-inflammation index.

ROC analysis demonstrated that the AUC for SII in predicting CTEPH was 0.797 (95% CI: 0.682–0.912, *P* < .001), and the optimal cutoff value was 626.7, with a specificity of 73% and a sensitivity of 69%. (Fig. 1).

## 5. Discussion

In this study, our findings indicated that SII was significantly elevated in patients with a history of first unprovoked acute PE who developed CTEPH, and that SII was an independent determinant of CTEPH in this patient group.

Previous studies have indicated that inflammation not only plays a role in the pathophysiology of CTEPH but also contributes to disease progression, pointing to a detrimental impact of chronic inflammation in patients with CTEPH.^[5,17]^ In a study by Gok et al,^[18]^ the SII level was shown to be significantly higher in patients with acute PE than in those without, and this elevation progressively increased from nonmassive to massive acute PE. In our study, while there was no statistically significant difference in the SII between the 2 groups in the acute period, the SII was found to be significantly higher after 3 months in the group that developed CTEPH. This suggests that SII may not serve as an early risk prediction marker at the time of acute PE presentation, but may instead reflect ongoing disease processes during follow-up.

In nearly 90% of acute PE cases, pulmonary thromboemboli naturally resolve completely or leave minimal residual material, with pulmonary hemodynamics returning to normal within 30 days.^[19]^ Previous studies in CTEPH patients using C-reactive protein levels have provided some evidence suggesting that thrombus resolution may be altered by inflammation.^[20,21]^ According to the “inflammatory thrombosis” theory, inflammation promotes abnormal proliferation of new thrombi on the endothelial surface, which subsequently transform into fibrotic tissue.^[22]^ Moreover, because thromboembolic resolution does not occur in patients with CTEPH, histopathological examination of pulmonary endarterectomy samples has revealed a greater accumulation of inflammatory cells in thrombotic lesions than in recanalized regions. Neutrophils, a major cellular component of the SII, were also identified in these samples. Considering the role of neutrophils in persistent inflammation associated with advanced vascular lesions,^[6]^ this may explain the significantly elevated neutrophil counts observed in CTEPH patients with post-3-month follow-up data in our study. Furthermore, elevated levels of neutrophil extracellular traps have been detected in the plasma of patients with CTEPH and are thought to contribute to the progression of chronic thrombosis in CTEPH by promoting thrombofibrosis and inflammation.^[23,24]^

In our study, platelet count, a major cellular component of the SII, was significantly higher in CTEPH patients than in non-CTEPH patients. A study by Yaoita et al,^[25]^ reported that in contrast to non-PH patients, individuals with CTEPH exhibited elevated levels of platelet activation and demonstrated increased hyperreactivity to thrombin stimulation. Specifically, upon ex vivo thrombin stimulation, platelets from CTEPH patients showed higher surface expression of *P* selectin and greater PAC-1 binding than those from non-PH patients. This hyper-responsiveness of platelets may exacerbate inflammatory reactions in the endothelial cells of the pulmonary artery, thereby contributing to the etiology and progression of CTEPH.

The prognostic utility of SII as a robust thrombo-inflammatory biomarker has been increasingly recognized across a wide spectrum of cardiovascular pathologies. Studies have demonstrated that elevated SII is independently associated with major adverse cardiovascular and cerebrovascular events in high-risk populations, such as patients with diabetes and acute coronary syndrome undergoing percutaneous coronary intervention, as well as those with acute coronary syndrome and concomitant chronic kidney disease.^[26,27]^ In the context of heart failure, SII has been identified as a significant predictor of mortality and long-term adverse outcomes. Specifically, Hayiroğlu et al highlighted that higher SII levels are independently linked to 10-year mortality and appropriate shock therapy in patients with heart failure with reduced ejection fraction undergoing implantable cardioverter defibrillator implantation.^[28]^ Furthermore, in patients with non-ST-elevation myocardial infarction, it was shown that SII serves as an independent predictor of both in-hospital and long-term mortality, exhibiting superior predictive performance compared to traditional laboratory ratios and correlating with established risk metrics like the thrombolysis in myocardial infarction and “History, ECG, Age, Risk, Troponin” scores.^[29]^ These findings underscore that SII effectively reflects a persistent “inflammatory burden” that contributes to adverse clinical outcomes even in chronic cardiovascular settings where overt inflammation may not be clinically apparent. Relatedly, other hematologic indices such as the monocyte-to-HDLC ratio and the neutrophil-to-lymphocyte and platelet ratio have been shown to reflect meaningful risk and to serve as useful biomarkers for assessing disease progression and recovery, reinforcing the concept that these hematologic markers can capture complex immune-thrombotic pathways.^[30,31]^

According to our results, SII was identified as an independent, significant determinant of CTEPH in multivariate logistic regression analysis. ROC analysis demonstrated an AUC of 0.797 (95% CI: 0.682–0.912, *P* < .0001) for the SII in predicting CTEPH, with an optimal cutoff value of 626.7, specificity of 73%, and sensitivity of 69%. Similarly, a recent study proposed the systemic inflammatory response index, a composite marker incorporating neutrophil, monocyte, and lymphocyte counts, as an indicator of systemic inflammation contributing to thrombofibrosis, and found it to be an independent predictor of clinical deterioration in patients with CTEPH.^[32]^ While our results identify SII measured at 3 months after the index PE as an independent determinant of CTEPH, a cautious interpretation of causality is warranted. As previously emphasized, despite the absence of a significant difference in acute-phase SII levels, significantly higher SII levels measured at 3 months after the index PE in patients who subsequently developed CTEPH suggest that sustained rather than transient inflammation may play a role in the pathobiological processes leading to CTEPH. Specifically, elevated SII may reflect the severity of the underlying thrombo-inflammatory process associated with the transition from acute PE to chronic PH. In this context, SII may be interpreted along 3 nonexclusive dimensions: as a potential risk stratification marker during follow-up, as a surrogate of disease severity reflecting thrombotic burden, and as an indicator of persistent thrombo-inflammatory activity during disease evolution. Nevertheless, whether this inflammatory activity represents a causal mechanism or a consequence of evolving disease remains unclear. Therefore, rather than being considered a true independent predictor of future risk, elevated SII may primarily represent a marker of persistent inflammatory activity and overall disease severity.

Our study has several limitations. First, the retrospective case-control design limits our ability to draw definitive temporal or causal conclusions regarding the relationship between SII and the development of CTEPH; therefore, the observed associations should be interpreted as correlational. In addition, due to the retrospective nature of the study, SII values could not be assessed dynamically over the course of the disease. Second, the relatively small sample size may limit the generalizability of our findings and may have reduced our ability to detect subtle predictors during the acute phase. In addition, no healthy control group was included, which may further limit the external validity of the results. Third, patients were not stratified according to the extent or location of pulmonary lesions. Another limitation is that we did not assess whether calculating the SII from multiple blood samples, rather than a single measurement, might have influenced the results. On the other hand, although CTEPH patients were already under clinical follow-up, all data for both CTEPH and non-CTEPH patients were collected retrospectively. This retrospective design minimizes the risk of differential selection, as all eligible patients were identified through predefined inclusion and exclusion criteria. Future prospective, multicenter studies with larger sample sizes are needed to validate our findings.

## 6. Conclusion

The SII may serve as a practical and readily accessible biomarker for predicting CTEPH during the follow-up of patients with a history of acute PE. Its moderate-to-high diagnostic performance suggests that the SII could be incorporated into routine clinical screening or utilized as part of a multimarker panel.

## Author contributions

**Conceptualization:** Hatice Solmaz.

**Data curation:** Burcu Uludag.

**Formal analysis:** Ayse Colak.

**Methodology:** Hatice Solmaz, Ayse Colak.

**Writing – original draft:** Hatice Solmaz.

**Writing – review & editing:** Hatice Solmaz.
